# A Digital Intervention to Promote Self-Management Self-Efficacy Among Community-Dwelling Individuals With Stroke: Pilot Randomized Controlled Trial

**DOI:** 10.2196/50863

**Published:** 2024-02-19

**Authors:** Zhaoying Li, Yating Lei, Quoc Bui, Olivia DePaul, Ginger E Nicol, David C Mohr, Sunghoon I Lee, Mandy W M Fong, Christopher L Metts, Stephanie E Tomazin, Alex W K Wong

**Affiliations:** 1 Division of Occupational Science and Occupational Therapy University of North Carolina School of Medicine Chapel Hill, NC United States; 2 Department of Occupational Therapy New York University New York, NY United States; 3 Institute for Informatics, Data Science & Biostatistics Washington University School of Medicine St. Louis, MO United States; 4 Memorial Hospital Belleville Belleville, IL United States; 5 Department of Psychiatry Washington University School of Medicine St. Louis, MO United States; 6 Department of Preventive Medicine, Feinberg School of Medicine Northwestern University Chicago, IL United States; 7 Center for Behavioral Intervention Technologies, Feinberg School of Medicine Northwestern University Chicago, IL United States; 8 Manning College of Information and Computer Sciences University of Massachusetts Amherst Amherst, MA United States; 9 Michigan Avenue Neuropsychologists Chicago, IL United States; 10 Department of Pathology and Laboratory Medicine Medical University of South Carolina Charleston, SC United States; 11 Center for Rehabilitation Outcomes Research Shirley Ryan AbilityLab Chicago, IL United States; 12 Department of Physical Medicine and Rehabilitation, Feinberg School of Medicine Northwestern University Chicago, IL United States; 13 Department of Medical Social Sciences, Feinberg School of Medicine Northwestern University Chicago, IL United States

**Keywords:** digital intervention, feasibility, mobile health, participation, rehabilitation, self-efficacy, self-management, stroke, technology, telehealth, telemedicine, text messaging

## Abstract

**Background:**

Digital interventions provided through smartphones or the internet that are guided by a coach have been proposed as promising solutions to support the self-management of chronic conditions. However, digital intervention for poststroke self-management is limited; we developed the interactive Self-Management Augmented by Rehabilitation Technologies (iSMART) intervention to address this gap.

**Objective:**

This study aimed to examine the feasibility and initial effects of the iSMART intervention to improve self-management self-efficacy in people with stroke.

**Methods:**

A parallel, 2-arm, nonblinded, randomized controlled trial of 12-week duration was conducted. A total of 24 participants with mild-to-moderate chronic stroke were randomized to receive either the iSMART intervention or a manual of stroke rehabilitation (attention control). iSMART was a coach-guided, technology-supported self-management intervention designed to support people managing chronic conditions and maintaining active participation in daily life after stroke. Feasibility measures included retention and engagement rates in the iSMART group. For both the iSMART intervention and active control groups, we used the Feasibility of Intervention Measure, Acceptability of Intervention Measure, and Intervention Appropriateness Measure to assess the feasibility, acceptability, and appropriateness, respectively. Health measures included the Participation Strategies Self-Efficacy Scale and the Patient-Reported Outcomes Measurement Information System’s Self-Efficacy for Managing Chronic Conditions.

**Results:**

The retention rate was 82% (9/11), and the engagement (SMS text message response) rate was 78% for the iSMART group. Mean scores of the Feasibility of Intervention Measure, Acceptability of Intervention Measure, and Intervention Appropriateness Measure were 4.11 (SD 0.61), 4.44 (SD 0.73), and 4.36 (SD 0.70), respectively, which exceeded our benchmark (4 out of 5), suggesting high feasibility, acceptability, and appropriateness of iSMART. The iSMART group showed moderate-to-large effects in improving self-efficacy in managing emotions (*r*=0.494), symptoms (*r*=0.514), daily activities (*r*=0.593), and treatments and medications (*r*=0.870), but the control group showed negligible-to-small effects in decreasing self-efficacy in managing emotions (*r*=0.252), symptoms (*r*=0.262), daily activities (*r*=0.136), and treatments and medications (*r*=0.049). In addition, the iSMART group showed moderate-to-large effects of increasing the use of participation strategies for management in the home (*r*=0.554), work (*r*=0.633), community (*r*=0.673), and communication activities (*r*=0.476). In contrast, the control group showed small-to-large effects of decreasing the use of participation strategies for management in the home (*r*=0.567), work (*r*=0.342, community (*r*=0.215), and communication activities (*r*=0.379).

**Conclusions:**

Our findings support the idea that iSMART was feasible to improve poststroke self-management self-efficacy. Our results also support using a low-cost solution, such as SMS text messaging, to supplement traditional therapeutic patient education interventions. Further evaluation with a larger sample of participants is still needed.

**Trial Registration:**

ClinicalTrials.gov 202004137; https://clinicaltrials.gov/study/NCT04743037?id=202004137&rank=1

## Introduction

People receive limited inpatient rehabilitation services after a stroke, with an average rehabilitation stay of 18.6 days [1]. Those with no major motor impairments (eg, neurologically mild stroke) are often discharged from acute care without rehabilitation [2,3]. Stroke survivors are at risk for developing depression [4], experiencing reduced quality of life [5], and having an increased chance of stroke recurrence [6,7]. Moreover, restricted participation in home, community, work, and social activities following stroke is common [8,9] and can last over 6 months [10]. Stroke survivors often manifest chronic neuropsychiatric symptoms (eg, fatigue, depressed mood, and cognitive dysfunction), which can impact their stroke recovery and delay or prevent a return to prestroke social roles [11]. Thus, learning strategies to manage poststroke symptoms and cope with challenges after transitioning back to community living is essential in stroke rehabilitation [9]. Self-management programs, also known as therapeutic patient education interventions [12], could help stroke survivors improve health management and participation in home, work, and community activities [11,13]. Most stroke self-management programs use a self-efficacy–building approach to promote and maintain active participation in home and community activities poststroke [14]. Improving self-efficacy to manage symptoms and chronic conditions ultimately leads to enhanced participation [11,13]. A systematic review of 22 studies (N=1761) investigated the influence of interventions supporting self-management skills on poststroke outcomes. Given the heterogeneity of the findings, no meta-analysis was conducted. However, the results showed that self-management interventions based on self-efficacy principles could improve the quality of life, depression, daily activities, and physical functioning in stroke survivors [15]. Targeting self-efficacy in managing symptoms and behaviors becomes a critical behavioral approach to addressing the long-term consequences of stroke [15,16].

Self-management interventions are well suited to mobile health (mHealth) technologies [17,18] as mHealth delivery methods offer several advantages, including increased access for individuals who live in rural areas or have limited transportation options. Additionally, mHealth technologies provide the potential for real-time monitoring and feedback, the ability to tailor intervention components to individualized needs, and the ability to reduce administration costs [19,20]. A meta-analysis of 14 randomized controlled trials (N=1597) focused on examining what theories were applied to the development of technology-based self-management interventions and investigating their effectiveness in improving depression, anxiety, fatigue, and self-efficacy for people with neurological disorders. The results showed that cognitive-behavioral and social-cognitive theories are the 2 most common theories used to develop technology-based self-management interventions in individuals with neurological disorders. In addition, cognitive-behavioral theory–based interventions were effective in enhancing self-efficacy and reducing depression, anxiety, and fatigue. In contrast, social-cognitive theory–based interventions were effective in reducing depression only [21]. In particular, this review found large effects in enhancing self-efficacy and reducing anxiety and moderate effects in reducing depression and fatigue. Although this meta-analysis showed promising results for neurological disorders, the study populations in these 16 studies did not include people after a stroke. Thus, research is needed to verify that this evidence applies to people after a stroke. To harness the benefits of the mHealth delivery, we developed a technology-supported self-management intervention, the interactive Self-Management Augmented Rehabilitation Technologies (iSMART) intervention, adapted from the face-to-face, stroke-focused psychoeducation program Improving Participation after Stroke Self-Management (IPASS) [11,13]. iSMART simplified the original IPASS psychoeducation sessions and added text messaging and behavioral coaching components [22]. We integrated SMS text messaging into iSMART because it is easily customized to individual needs and accessible to anyone with a cell phone [23,24]. Live health coaches, based on behavioral activation [25], supplement psychoeducation sessions to support intervention uptake and promote effective collaboration, negotiation, and motivation while encouraging individuals to take responsibility for their recovery and wellness by fostering healthy behaviors [26].

To test this novel intervention’s feasibility and potential benefits, this study aimed to (1) evaluate the acceptability, appropriateness, and feasibility of iSMART in individuals with stroke and (2) establish the preliminary effect size of iSMART in improving self-management self-efficacy in individuals after stroke. We hypothesized that (1) iSMART would be feasible to deliver and be acceptable to people with stroke and (2) iSMART would result in a moderate effect for improving poststroke self-management self-efficacy.

## Methods

### Design and Recruitment

We conducted a parallel, 2-arm, nonblinded, randomized controlled trial of 12-week duration. Participants were recruited from a stroke registry at a university-affiliated acute care hospital between January and March 2021. Using a random number generator guided by a biostatistician, participants were randomized to receive either the iSMART intervention or a manual of stroke rehabilitation (attention control). All participants in both groups continued receiving standard-of-care rehabilitation services their treating physicians recommended.

### Participants and Randomization

**Figure 1:**
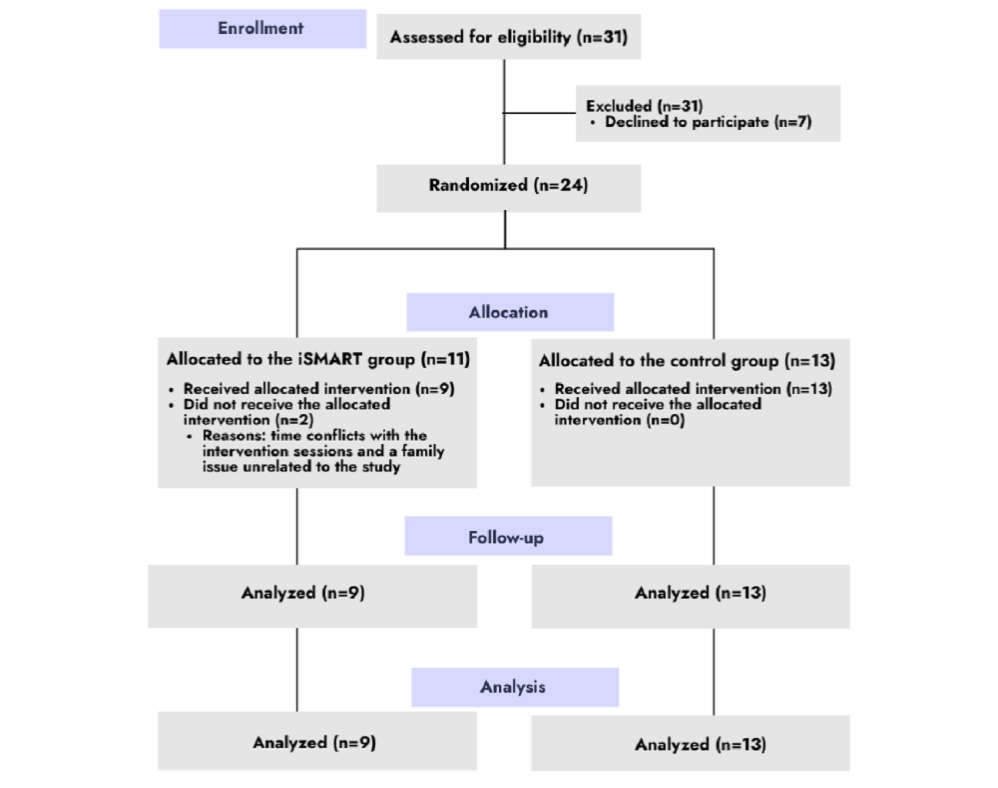
CONSORT (Consolidated Standards of Reporting Trials) flow diagram showing participant recruitment and completion. iSMART: interactive Self-Management Augmented by Rehabilitation Technologies.

Potential participants (N=31) were recruited between January 2021 and March 2021 based on the following inclusion and exclusion criteria. Inclusion criteria were (1) mild-to-moderate stroke (National Institutes of Health Stroke Scale scores ≤13) [27], (2) ischemic or hemorrhagic stroke, (3) aged 18 years or older, (4) English-speaking, (5) ≥3 months after stroke, (6) self-identified as having ≥1 chronic condition, and (7) mobile phone ownership. Exclusion criteria were (1) preexisting neurologic or psychiatric disorder (eg, dementia or schizophrenia), (2) severe poststroke cognitive impairment (Short Blessed Test score ≥9), (3) history of functional problems (Premorbid Modified Ranking Scale score ≥2) before the stroke, (4) severe aphasia (Boston Naming Test <10) [28], and (5) visual problems that make reading words on the device difficult. Of the screened individuals who had a stroke, 24 were randomized (CONSORT [Consolidated Standards of Reporting Trials] diagram; Figure 1).

### Procedures

#### Overview

This study was a remote clinical trial, that is, a clinical trial performed remotely, including the interaction between the experimenter and participant and the assessment of outcomes [29]. Study staff contacted potential participants from a stroke research registry at a university-affiliated hospital in the Midwestern United States to explore their interest in the study. After that, study staff sent participants a secure link through email or SMS through the REDCap (Research Electronic Data Capture; Vanderbilt University) [30] and scheduled video or phone sessions to assist participants in completing the consent form and screening test for eligibility. Eligible participants were randomly allocated to the iSMART or control groups using a random sequence computer-generated program to ensure allocation concealment. Neither study staff nor participants were masked for randomization assignments. Following consent, participants underwent a remote enrollment, at which iSMART participants were oriented to technologies used in the study (ie, the videoconferencing platform and SMS) by study staff. Study staff also obtained the phone’s operating system (Android or iOS) and linked the phone number to the web-based iSMART platform used to send and receive text messages from participants. Participants in both groups started their allocated interventions after all participants completed baseline testing. The intervention lasted for 3 months. After completing their allocated interventions, all participants completed a postintervention assessment. Participants in both groups continued to receive health services as prescribed by their clinicians. Participants in the iSMART group were compensated US $300 for completing the allocated intervention and outcome measures and data plan coverage. Participants in the control group were compensated US $120 for completing the allocated intervention and outcome measures. No messages were sent to participants in the control arm, so they were not compensated for data usage. The trial ended in June 2021.

#### The iSMART Intervention

The iSMART was a 12-week, technology-supported, coach-guided, self-management intervention comprising 3 components: psychoeducation, behavioral coaching, and text messaging. A licensed occupational therapist served as the coach in this study. The psychoeducation component was built upon the Social Cognitive Theory [31] and the person-environment-occupation-performance model [32] and implemented through weekly, 2.5-hour sessions in a group videoconferencing format. These sessions focused on teaching participants self-management strategies, including problem-solving, decision-making, positive thinking, communication, and accommodation, for managing symptoms and supporting participation in home, work, community, and social activities.

The coaching component was built on behavioral activation theory and modified from the *Revised Treatment Manual of the Brief Behavioral Activation Treatment for Depression* [25]. It was implemented weekly in 0.5-hour sessions in a one-to-one videoconferencing format. Individual coaching sessions engaged participants in collaborative goal setting with the coach to identify values and select personal activity goals from 25 predefined goals. The coach then entered the selected goals into the web-based iSMART platform so participants could receive messages customized to their chosen goals. These goals target improving participation in different life areas, including daily responsibilities, relationships, interests and recreation, education and career, and mind, body, and spirituality derived from the behavioral activation manual [25].

**Figure 2:**
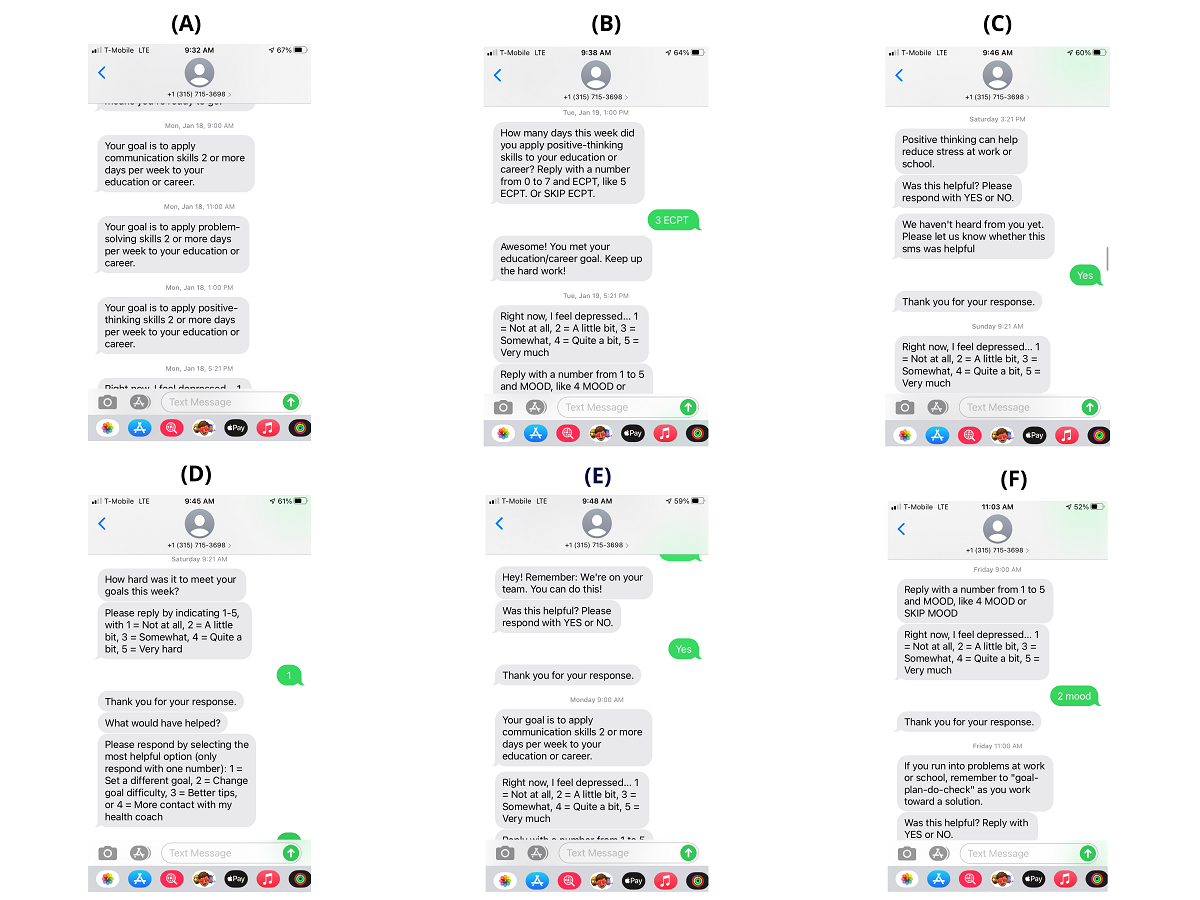
Screenshots of different types of messages. (A) goal reminder, (B) goal monitoring, (C) self-management tip, (D) ecological needs assessment, (E) general motivation, and (F) mood monitoring.

The text messaging component was adapted from previous studies, with effectiveness demonstrated in hospital workers [33,34] and adults with severe mental illness [35]. We adapted and pretested text messages with the planning group members, intending to increase the uptake by individuals with stroke (details in the next paragraph). Text messages were sent following the predefined schedules, including goal reminders (delivered on Mondays), goal monitoring (Tuesdays), mood monitoring (daily), self-management tips (Thursdays to Saturdays), ecological needs assessment (Saturdays), and motivational messages (Sundays). Figure 2 provides snapshots of these messages.

We formed a planning group, including 2 stroke rehabilitation clinicians, a stroke survivor, a technologist, and a self-management expert, to guide the intervention adaptation using a systematic intervention-mapping process [22,36]. During this adaptation process, we applied the behavior change wheel [37] and behavioral change technique taxonomy [38] to specify strategies that help individuals change self-management behaviors. Specifically, we identified 7 behavioral determinants most likely to affect the intervention goal and outcomes, including knowledge, behavioral regulation, skills, self-efficacy, motivation, negative and positive affect, and social and environmental support. We also identified the mechanisms of action (eg, beliefs about capabilities, values, knowledge, and motivation) most likely to affect the selected behavioral determinants. We then used the linkage table published by Carey et al [39] to match the behavioral change techniques (eg, information about health consequences, information about social and environmental consequences, instructions on how to perform the behavior, and feedback on behavior) to each of the mechanisms of action. Finally, to ensure iSMART should be applied to the selected behavioral change techniques, we developed a set of empirically supported strategies and integrated these strategies into different parts of the 3 treatment components. Details of the intervention development of iSMART, including the theoretical framework, mechanisms of action, behavioral change techniques, and the set of empirically supported strategies, are described elsewhere [22].

#### Control Intervention

Participants in the control group received a study-specific manual comprising stroke-specific information based on resources from the American Stroke Association and the Canadian Stroke Association. Manual content includes stroke overview, stroke prevention, rehabilitation, fatigue, weight management, fitness, medication, sleep, balance, healthy eating, emotional changes, social support, home modifications, and return to work or school. This study staff made telephone calls once a week to ask if participants had any problems while reading the manual and encouraged participants to read through the manual. The study staff did not deliver any iSMART content.

### Outcome Measures

#### Feasibility Measures

Rates of retention and engagement were automatically recorded through the web-based iSMART platform. We defined retention as the rate at which participants completed or remained in the study and engagement as the rate at which participants responded to text messages. We defined retention and engagement rates as ≥80%, based on a previous technology intervention that showed participants who achieved these criteria demonstrated better outcomes [35]. The project found that participants who met the criteria would demonstrate better target health outcomes. Participants also completed three 4-item implementation measures postintervention: the Feasibility of Intervention Measure (FIM), the Acceptability of Intervention Measure (AIM), and the Intervention Appropriateness Measure (IAM) [40]. Weiner et al [40] found that these measures had strong structural validity with .89 for FIM, .85 for AIM, and 0.91 for IAM and test-retest reliability with .88 for FIM, .83 for AIM, and .87 for IAM. However, no discriminant validity of these measures was studied [40]. We defined the benchmark for high feasibility, acceptability, and appropriateness as the mean score of 4 (out of 5) on the FIM, AIM, and IAM.

#### Self-Efficacy Measures

Participants completed the Participation Strategies Self-Efficacy Scale (PS-SES) and the Patient-Reported Outcomes Measurement Information System’s Self-Efficacy (PROMIS-SE) for managing chronic conditions at baseline and postintervention. PS-SES is a 35-item measure to assess self-efficacy in using participation strategies to manage home, work, community, and communication [41]. Lee et al [41] found that the Cronbach α coefficients of internal consistency of PE-SES were high (α=.884 to .926).

PROMIS-SE consists of five 4-item short forms to assess self-efficacy for managing daily activities, medications, treatment, symptoms, emotions, and social interactions [42]. Confirmatory factor analyses confirmed the multidimensional structure of the PROMIS-SE.

### Data Analysis

Participants who completed the intervention were selected for data analyses, as we did not compute any missing values of outcomes for those who did not complete the study. Demographic characteristics between the 2 groups were evaluated using Fisher exact tests or Wilcoxon rank sum tests. Considering the small sample size of this study, we computed nonparametric analyses with median scores of FIM, AIM, and IAM and self-efficacy measures. We reported both mean and median scores for resolution purposes.

We compared retention and engagement rates and the FIM, AIM, and IAM scores of the iSMART intervention with the predefined benchmarks. We conducted Wilcoxon rank sum tests to evaluate any differences between the groups on FIM, AIM, and IAM scores. To establish the effect sizes for change in self-efficacy, we computed change scores from baseline to postintervention. We then compared the change scores between the 2 groups using Wilcoxon rank sum tests. Due to the small size, any demographic differences between groups at baseline may have artificially inflated the group difference in study outcomes. Thus, we also examined any significant changes for each group using Wilcoxon signed rank tests. We used effect sizes to interpret the intervention effect instead of statistical significance (ie, *P*≤.05) [43]. We defined small effects if 0.1≤*r*<0.3, moderate effects if 0.3≤*r*<0.5, and large effects if *r*≥0.5 [44]. We reported effect sizes as they were independent of sample size so that we could express the size of an intervention effect regardless of the size of the study [45].

### Ethical Considerations

All participants provided informed consent. The ethics committees of Washington University (202004137) and Northwestern University (STU00215743) reviewed and approved this study. We registered the study at ClinicalTrials.gov (202004137). We reported this study adhering to the CONSORT statement [46,47].

## Results

### Participants

Participant flow is presented in Figure 1. A total of 31 participants were screened, 24 were randomized, and 22 (iSMART: n=13 and control: n=9) completed the study. Table 1 shows the baseline characteristics of the participants.

**Table 1:** Clinical and demographic information of the participants.

Variables					Overall (n=24)		Control (n=13)			iSMART^a^ (n=11)			*P* value^b^	
Age (years), mean (SD)					59 (12)		57 (12)			62 (11)			.35	
**Sex, n (%)**														>.99
	Male			14 (58)		8 (62)			6 (55)					
	Female			10 (42)		5 (38)			5 (45)					
**Marital status, n (%)**														>.99
	Married or cohabitating			13 (54)		7 (54)			6 (55)					
	Separated, divorced, or widowed			7 (29)		4 (31)			3 (27)					
	Single			4 (17)		2 (15)			2 (18)					
**Total household income (US $), n (%)**														.18
	0 to 14,999			3 (12)		0 (0)			3 (27)					
	15,000 to 34,999			5 (21)		2 (15)			3 (27)					
	35,000 to 54,999			4 (17)		4 (31)			0 (0)					
	55,000 to 74,999			3 (12)		2 (15)			1 (9.1)					
	75,000 or more			7 (29)		4 (31)			3 (27)					
	Do not wish to answer			2 (8.3)		1 (7.7)			1 (9.1)					
**Premorbid disability (Modified Rankin Scale), n (%)**														.77
			No symptoms	20 (83)		11 (85)			9 (82)					
			No significant disability	3 (12)		2 (15)			1 (9.1)					
			Slight disability	1 (4.2)		0 (0)			1 (9.1)					
Stroke severity (NIH^c^ Stroke Scale), mean (SD)					3.5 (4.2)		1.8 (3.1)			5.5 (4.7)			.06	
**Residential status, n (%)**														.21
			Alone	8 (33)		6 (46)			2 (18)					
			With others	16 (67)		7 (54)			9 (82)					
**Financial responsibilities, n (%)**														.46
			Dependent	23 (96)		13 (100)			10 (91)					
			Primary or partial responsibility	1 (4.2)		0 (0)			1 (9.1)					
**Race, n (%)**														.68
	Black			9 (38)				4 (31)			5 (45)			
	White			15 (62)		9 (69)			6 (55)					
**Stroke diagnosis, n (%)**														>.99
	Hemorrhagic			4 (17)				2 (15)			2 (18)			
	Ischemic			20 (83)		11 (85)			9 (82)					
**Stroke side, n (%)**														.75
		Bilateral		1 (4.2)		0 (0)			1 (9.1)					
		Left		7 (29)		3 (23)			4 (36)					
		Right		9 (38)		6 (46)			3 (27)					
		Unknown		7 (29)		4 (31)			3 (27)					
Time since stroke (days), mean (SD)					1245 (1079)		957 (1059)			1585 (1048)			.09	
Education (years), mean (SD)					15 (3)		14 (3)			15 (3)			.55	
Number of the previous stroke, mean (SD)					2 (4)		2 (2)			3 (5)			.18	

^a^iSMART: interactive Self-Management Augmented by Rehabilitation Technologies

^b^Wilcoxon rank sum test; Fisher exact test.

^c^NIH: National Institutes of Health.

### Feasibility Measures

#### Retention and Engagement

A total of 2 participants in the iSMART group withdrew from the study, resulting in a retention rate of 82% (9/11) that exceeded the predefined benchmark. Reasons for withdrawal included (1) time conflicts with the group sessions and (2) a family issue unrelated to the intervention. The engagement (SMS text message response) rate across all participants was 76%, ranging from 22% to 96%. Although the overall engagement rate was slightly below the predefined benchmark, only 2 out of 9 participants had response rates less than 80% (ie, 22% and 49%).

#### Feasibility, Acceptability, and Appropriateness

The mean scores of FIM, AIM, and IAM for the iSMART participants were 4.11 (SD 0.61), 4.44 (SD 0.73), and 4.36 (SD 0.70), respectively, which met our benchmarks, suggesting high feasibility, acceptability, and appropriateness of the iSMART intervention (Table 2). Participants in the iSMART group rated higher FIM, AIM, and IAM scores than those in the control group, with a moderate effect for feasibility (*r*=0.449; *P*=.04) and large effects for acceptability (*r*=0.505; *P*=.02) and appropriateness (*r*=0.540; *P*=.01).

**Table 2:** Feasibility, acceptability, and appropriateness measures between the interactive Self-Management Augmented by Rehabilitation Technologies (iSMART) and control groups.

Measures	Control (n=13)			iSMART (n=9)			Wilcoxon statistic		Effect size
	Mean (SD)	Median (IQR)	Mean (SD)		Median (IQR)				
FIM^a^	3.48 (0.65)	3 (3, 4)	4.11 (0.61)		4 (4, 4.25)	28.5		0.449	
AIM^b^	3.60 (0.66)	3.5 (3, 4)	4.44 (0.73)		5 (4, 5)	24.5		0.505	
IAM^c^	3.54 (0.63)	3.5 (3, 4)	4.36 (0.70)		4.25 (4, 5)	22		0.540	

^a^FIM: Feasibility of Intervention Measure.

^b^AIM: Acceptability of Intervention Measure.

^c^IAM: Intervention Appropriateness Measure.

### Self-Efficacy Measures

**Figure 3:**
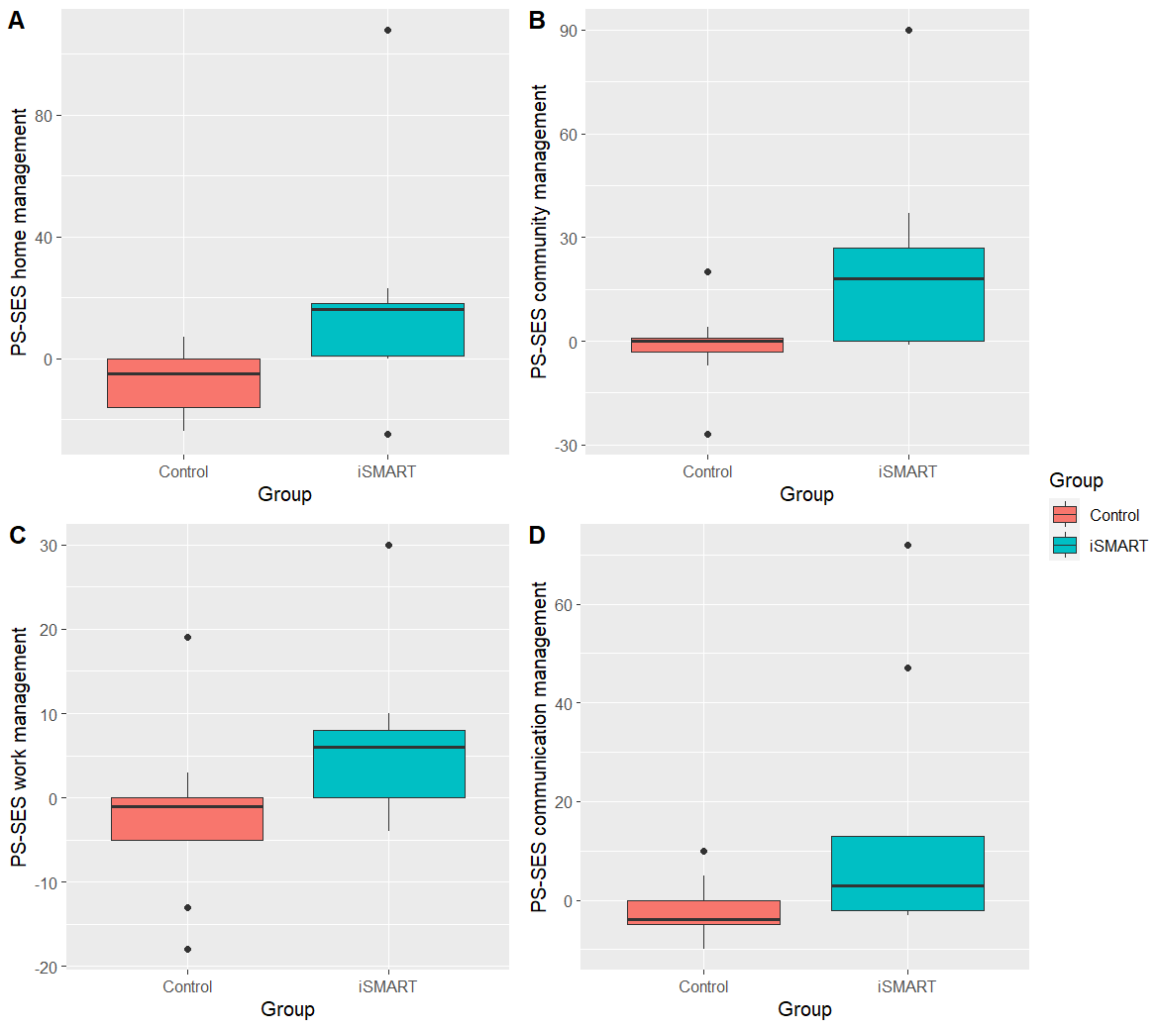
Changes in Participation Strategies Self-Efficacy Scale (PS-SES) scores after intervention. iSMART: interactive Self-Management Augmented by Rehabilitation Technologies.

**Figure 4:**
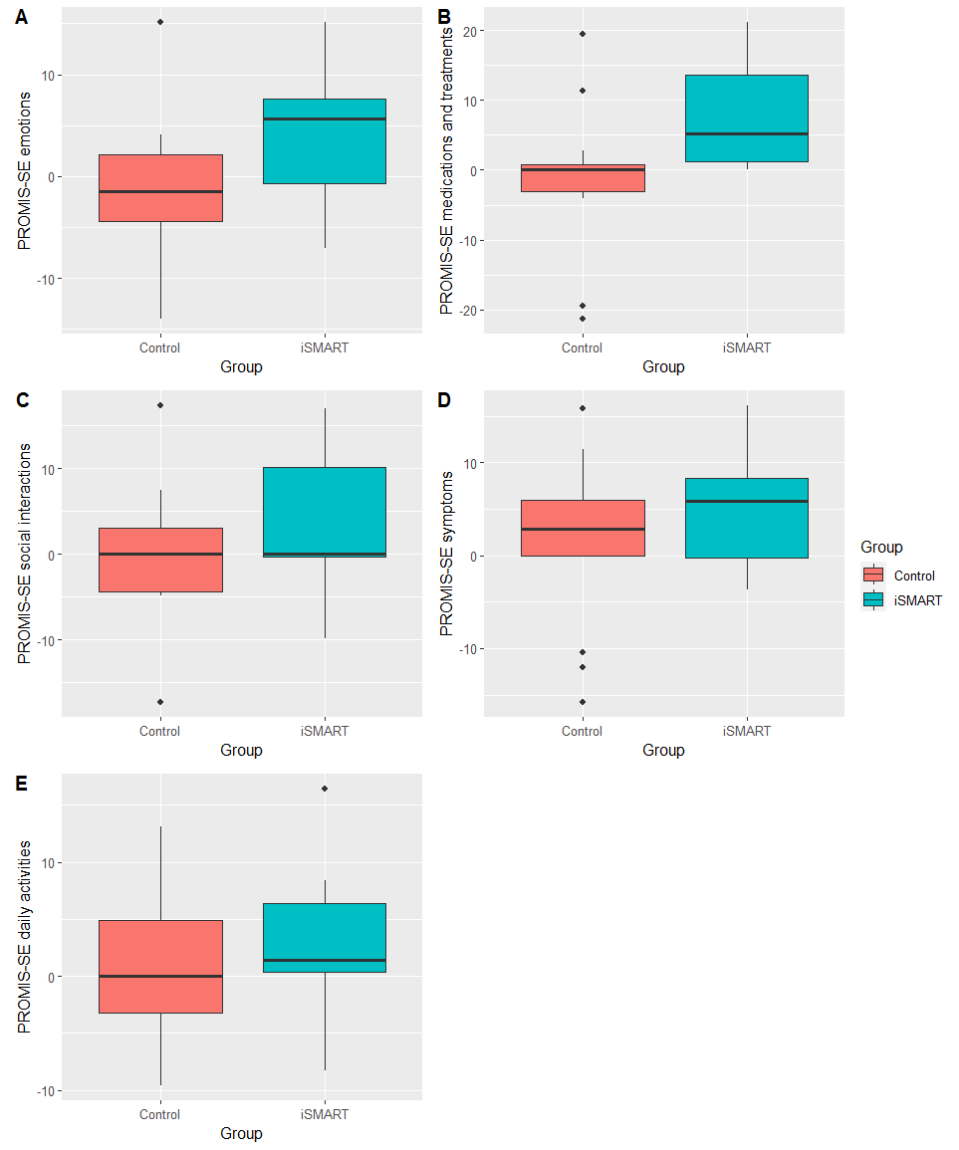
Changes in Patient-Reported Outcomes Measurement Information System’s Self-Efficacy (PROMIS-SE) scores after intervention. iSMART: interactive Self-Management Augmented by Rehabilitation Technologies.

Figures 3 and 4 show the PS-SES and PROMIS-SE change scores, illustrating significantly greater improvements in the iSMART group than in the control group. Table 3 shows the between-group effect sizes. All between-group effects were favorable to the iSMART group. PS-SES home management (*r*=0.571; *P*=.008), PS-SES community management (*r*=0.500; *P*=.02), and PROMIS-SE medications and treatments (*r*=0.506; *P*=.02) showed large effects. PS-SES work (*r*=0.464; *P*=.03), PS-SES communication management (*r*=0.478; *P*=.03), and PROMIS-SE emotions (*r*=0.313; *P*=.15) showed moderate effects.

**Table 3:** Pre- and postintervention self-efficacy scores between the control and interactive Self-Management Augmented by Rehabilitation Technologies (iSMART) groups.

Outcome measures		Control (n=13)							iSMART (n=9)							Between-group	
		Pre, mean (SD)	Post, mean (SD)	Pre, median (IQR)	Post, median (IQR)	*W*	*r*	Pre, mean (SD)		Post, mean (SD)	Pre, median (IQR)	Post, median (IQR)	*W*	*r*	*W*		*r*
**PS-SES^a^**																	
	Home management	105 (12.6)	97.5 (16.3)	106 (96, 113)	92 (82, 113)	47.5	.567	83.6 (38.5)		102 (21.5)	102 (57, 114)	115 (86, 120)	7	.554	18.5		.571
	Community management	82.8 (16.5)	81.3 (17.8)	82 (77, 100)	80 (72, 95)	35.5	.215	60.7 (36)		82.8 (20.8)	43 (35, 99)	98 (70, 100)	3	.673	23.5		.500
	Work management	60.6 (10.8)	58.8 (9.5)	65 (56, 68)	62 (51, 68)	39	.342	46.8 (19.1)		53.2 (18.3)	47 (36, 63)	56 (38, 70)	4	.633	26		.464
	Communication management	67.4 (15)	65.5 (14.6)	72 (66, 79)	71 (56, 76)	55.5	.379	52.9 (26.7)		67.9 (15.3)	62 (30, 77)	74 (60, 80)	7	.476	25		.478
**PROMIS-SE^b^**																	
	Emotions	47.1 (9.1)	46.1 (8.6)	49.6 (38.8, 53.2)	46.1 (38.5, 51.6)	58.5	.252	49.1 (7.8)		53.1 (7.6)	49.6 (41.0, 53.9)	51.5 (48.2, 55.3)	10	.494	36.5		.313
	Medications and treatments	46.1 (8.7)	45.1 (9.1)	43.5 (40.4, 50.4)	41.1 (38.8, 50.4)	42.5	.049	43.2 (9.9)		51.5 (10)	41.1 (37.1, 47.3)	55.5 (44.0, 60.6)	0	.870	23		.506
	Social interactions	44 (8.5)	44.3 (9.4)	42.5 (37.3, 48.4)	42.5 (38.8, 53.0)	36.5	.049	49.9 (10.1)		52.8 (7.5)	49.7 (42.5, 59.8)	52.9 (48.7, 59. 8)	6	.182	48.5		.143
	Symptoms	49.4 (8.8)	51.1 (8.1)	49 (44.8, 52.8)	47.7 (45.3, 57.2)	28.5	.262	49.7 (7.5)		55.1 (6.9)	48.8 (46.9, 53.7)	54.6 (50.0, 63.5)	6	.514	50		.121
	Daily activities	47.7 (8.2)	49 (6.7)	47.7 (42.7, 51.2)	46 (43.4, 54.8)	32	.136	46.7 (9.7)		49.9 (8.9)	44.4 (37.8, 53.3)	52.5 (42.1, 55.7)	6	.593	44.5		.199

^a^PS-SES: Participation Strategies Self-Efficacy Scale.

^b^PROMIS-SE: Patient-Reported Outcome Measurement Information System’s Self-Efficacy.

Table 3 further shows the within-group effect sizes. The iSMART showed moderate-to-large effects of increasing the use of participation strategies for management in the home (*r*=0.554; *P*=.14 [large]), work (*r*=0.633; *P*=.06 [large]), community (*r*=0.673; *P*=.04 [large]), and communication activities (*r*=0.476; *P*=.14 [moderate]). In contrast, the control group showed small-to-large effects of decreasing the use of participation strategies for management in the home (*r*=0.567; *P*=.05 [large]), work (*r*=0.342; *P*=.26 [moderate]), community (*r*=0.215; *P*=.44 [small]), and communication activities (*r*=0.379; *P*=.21 [moderate]).

In addition, the iSMART showed moderate-to-large effects of increasing self-efficacy in managing emotions (*r*=0.494; *P*=.16 [moderate]), symptoms (*r*=0.514; *P*=.11 [large]), daily activities (*r*=0.593; *P*=.11 [large]), and treatments and medications (*r*=0.870; *P*=.01 [large]), except a small effect of increasing self-efficacy in managing social interactions (*r*=0.182; *P*=.40). In contrast, the control group showed small effects of decreasing self-efficacy in managing emotions (*r*=0.252; *P*=.38), symptoms (*r*=0.262; *P*=.43), daily activities (*r*=0.136; *P*=.61), and treatments and medications (*r*=0.049; *P*=.81), except no change in self-efficacy in managing social interactions (*r*=0.049; *P*=.88).

## Discussion

### Principal Findings

This study evaluated the feasibility and established preliminary effect sizes of iSMART, an mHealth intervention for improving self-efficacy for chronic stroke management, in a group of community-dwelling stroke survivors. Our results showed that iSMART is feasible and acceptable for mild-to-moderate chronic stroke survivors. Participants also showed moderate improvements in most self-efficacy measures after completing the iSMART.

### Previous Works and Study Implications

We observed sufficient retention (82%) and engagement (SMS text message response) rates (76%) in the iSMART group. In addition, the iSMART group showed greater ratings than the control group on all 3 implementation measures, suggesting that iSMART is a feasible self-management program for stroke survivors. The iSMART had a similar retention rate to those reported in mHealth interventions for pediatric weight management (78%) [48], antiretroviral therapy (85%) [49], and tuberculosis treatment (87%) [49]. The text message response rate was similar to other mHealth interventions targeting behavior changes in neuropsychiatric conditions. Suffoletto et al [50] reported 74% to 97% messaging response rates in an education and behavioral support intervention using text messages to assess daily symptoms and provide support to adults with mild traumatic brain injury. Although we found that a larger portion of the iSMART participants met the engagement criteria (>80%), 2 out of 9 participants had response rates less than 80% (ie, 22% and 49%). The wide range of engagement was commonly found in other technology-based interventions for stroke survivors. For example, Guidetti et al [51] developed a technology-supported intervention for stroke survivors in Sweden and Uganda and stated that participants responded to 44% to 100% (mean 78%) of the text messages they received. A recent study of mHealth weight management intervention in adults with mental illness from which the iSMART was derived found that participants who met the criteria (>80% of text responses) in the first month of intervention had greater weight loss than those who did not [35]. These results suggest that future technology-based interventions may enhance intervention responses and effectiveness by increasing participants’ engagement up to the criteria that may maximize health and rehabilitation outcomes. Future studies are needed to formally test the engagement criteria and examine their relationships with treatment responses and outcomes for iSMART in stroke survivors.

Our findings indicated that iSMART yielded moderate-to-large effects in improving self-efficacy in using participation strategies for home, work, community, and communication management. Future interventions in improving participation outcomes following a stroke should make it a key behavioral target, given its beneficial mediatory effect on mobility and participation [52]. Participants who completed the iSMART intervention showed moderate-to-large effects of increasing self-efficacy in managing emotions, symptoms, daily activities, and treatments and medications. In contrast, the control intervention only yielded small effects. The beneficial effects of the iSMART intervention are consistent with other technology-supported self-management interventions that were effective in increasing self-efficacy and perceived participation in everyday life among stroke survivors [51,53]. This study also observed that mHealth delivery might amplify treatment effects. Compared to a nontechnology-based self-management intervention (ie, IPASS) that the iSMART was derived from, the SMART showed superior effects than the IPASS [11]. Nevertheless, because this study had a small sample (N=22), interpretations of these results should be very cautious. A future study using a larger sample size and using the face-to-face self-management program as a control is warranted to test the additional benefit of mHealth delivery of self-management interventions.

### Limitations and Future Directions

This study had several limitations. We did not conduct the intent-to-treat analysis in this pilot study. The intent-to-treat analysis has been considered the standard approach to randomized controlled trial analyses [54]. A future, definitive trial will complete this analysis to avoid biased estimates. In addition to the constraints associated with a small sample size, participants were recruited from a single institution, restraining the generalizability of the findings. We found a trend toward statistical significance for greater stroke severity and longer time since stroke in the iSMART group at baseline than the control group, which may have artificially inflated the difference between groups on study outcomes. For this feasibility study, we examined the intervention score changes using within-group models to avoid this potential bias and found results favoring the iSMART group. Nevertheless, future, and larger-scale studies are needed to examine if these factors were potential covariates affecting the treatment outcomes. We used 3 implementation measures to examine the treatment’s acceptability, appropriateness, and feasibility. Notably, these measures were fairly correlated, and their discriminant validity was not thoroughly tested. Thus, future research would benefit from further exploration of the discriminant validity of these constructs. This study did not collect information on how social support, built environment, technology access, and other environmental barriers impact intervention engagement in individuals with neuropsychiatric conditions, including stroke [55,56]. Future studies should examine whether these barriers mediate or modulate the impact of iSMART on poststroke outcomes.

Future research should consider the co-design approach when designing or adapting digital interventions to increase participant retention and engagement. Co-design is a process in which targeted end users and other relevant stakeholders’ partner with the research team to work together in all aspects of intervention development, testing, and dissemination [57]. Co-designed digital interventions are more effective than traditional approaches, where researchers and clinicians primarily design interventions [58]. This approach is particularly beneficial when collaborating with underrepresented and minority communities because the co-design allows for conceptual or tool redevelopments and refinements based on the social, linguistic, and cultural needs of partnership groups [59]. Future studies of iSMART will need to engage more stroke survivors and caregiver stakeholders in user-centered design activities, especially those from underserved communities, to identify which characteristics of the intervention, individual users, and the care environment best facilitate iSMART implementation and effectiveness [60].

This study only examined the effect of iSMART on self-efficacy over 12 weeks. Future studies are warranted to examine the long-term impact on self-efficacy and other disability outcomes, such as the reintegration of everyday living, quality of life, and perceived recovery in stroke survivors. iSMART included three intervention components. While considering all components together as a complex intervention, we found this intervention to have adequate feasibility and positive initial effects. A specific approach, the multiphase optimization strategy framework [61], has been used to test the performance of individual intervention components in the development of technology-supported interventions such as weight loss [62], palliative care [63], and physical activity promotion [64]. A future study is needed to identify the iSMART components (main effects or interactions) that contribute meaningfully to improvement in intervention engagement and health outcomes in people after stroke. Future research may test the multiphase optimization strategy approach to identify if all or some intervention components are needed to optimize the iSMART intervention.

### Conclusions

This study provides preliminary evidence to support the feasibility of delivering iSMART, a technology-supported self-management intervention to help stroke survivors increase self-efficacy for managing chronic conditions and supporting home, work, and community participation. Our findings support using a low-cost solution, such as text messaging, to supplement traditional therapeutic patient education interventions. More research is needed to provide more robust efficacy data to support the benefits of the iSMART intervention.

## Appendix

Multimedia Appendix 1 CONSORT-eHEALTH (V 1.6.1).

## Abbreviations

AIM: Acceptability of Intervention Measure
CONSORT: Consolidated Standards of Reporting Trials
FIM: Feasibility of Intervention Measure
IAM: Intervention Appropriateness Measure
IPASS: Improving Participation after Stroke Self-Management
iSMART: interactive Self-Management Augmented by Rehabilitation Technologies
mHealth: mobile health
NIH: National Institutes of Health
PROMIS-SE: Patient-Reported Outcomes Measurement Information System’s Self-Efficacy
PS-SES: Participation Strategies Self-Efficacy Scale
REDCap: Research Electronic Data Capture

## Data Availability

The data generated and analyzed during this study cannot be sufficiently deidentified and, therefore, cannot be made publicly available due to ethical considerations. Deidentified data can be made available for further research from the corresponding author on reasonable request.
